# Familial hemiplegic migraine type 2: a case report of an adolescent with ATP1A2 mutation

**DOI:** 10.3389/fneur.2024.1339642

**Published:** 2024-02-06

**Authors:** Hui Zhang, Li Jiang, Yuqi Xian, Sen Yang

**Affiliations:** ^1^The Fifth People’s Hospital of Chengdu, Chengdu, China; ^2^The Fifth People’s Hospital Affiliated to Chengdu University of Traditional Chinese Medicine, Chengdu, China

**Keywords:** ATP1A2, familial hemiplegic migraine, adolescent, hemiplegia, case report

## Abstract

This study presents a case report of a male adolescent diagnosed with familial hemiplegic migraine type 2 (FHM2), an autosomal dominant inheritance disorder caused by ATP1A2 mutation. We report the patient who presented with headache, aphasia, and left-sided weakness. Cerebrovascular disease and various infectious agents were unremarkable during the patient’s extended hospital stay. Our case revealed that brain hyperperfusion in familial hemiplegic migraine (FHM) persists over an extended duration, and despite the disease being in a state of recovery, enhanced brain magnetic resonance imaging (MRI) continues to exhibit hyperperfusion. A genetic testing was performed which revealed a mutation in the FHM2 gene (c.1133C > T). The patient has been followed for 3 years after hospital discharge. The boy suffered four episodes of hemiplegia and multiple episodes of headaches, and gradually developed seizures and cognitive impairment. It is advisable to consider FHM as a potential diagnosis for patients presenting with typical symptoms such as recurrent paroxysmal headaches and limb activity disorders.

## Introduction

Migraine is a complex neurological disorder that affects 11% of the adults and 5% of children worldwide (1, 2). FHM is an uncommon autosomal dominant form of migraine characterized by a unique aura (3). The International Classification of Headache Disorders (ICHD-3) diagnostic criteria for FHM are as follows: A. at least two attacks, B. the presence of a reversible motor deficit, C. at least two of the following four characteristics: 1.at least one aura symptom spreads gradually over ≥5 min, and/or two or more symptoms occur in succession, 2. each individual aura symptom lasts 5–60 min, 3. at least one aura symptom is unilateral, 4. the aura is accompanied, or followed within 60 min, by headache, D. similar episodes in relatives, and E. subjects with related diseases were excluded (4). Three specific mutations in causative genes have been identified: CACNA1A (which encodes the subunit of the voltage-gated Ca2+ channel CaV2.1), ATP1A2 (which encodes the α2-subunit of the Na+/K + -ATPase), and SCN1A (which encodes the alpha subunit of a voltage-gated neuronal sodium channel) (5–7). In this report, we present the case of a 13-year-old adolescent patient with an undocumented novel mutation and multiple imaging findings associated with FHM2.

## Case report

A 13-year-old male patient presented to our department with symptoms of headache, blurred vision, and left-sided weakness. Prior to his admission, the patient experienced a brief episode of blurred vision, followed by a severe right-sided headache accompanied by vomiting. Eventually, the patient developed difficulty moving his left side. Neurologic examination revealed drowsiness, restlessness upon stimulation, dysphagia, dysarthria, left-sided facial nerve palsy, left visual field defect, and a muscle strength of grade 1 in the left upper limb and grade 2 in the left lower limb. The boy experienced a febrile convulsion at the age of 6 months. Leading to hospitalization for fever, headache, and general convulsion at the ages of 4 and 5, respectively, with a subsequent coma lasting 5 to 6 days. He received a diagnosis of viral encephalitis and epilepsy. However, over the next 5 years, his electroencephalogram (EEG) showed no abnormalities. In his familial history, his grandmother has a history of recurrent headaches but no occurrences of hemiplegia.

Upon admission, the boy’s body temperature was within the normal range. Nevertheless, he developed a fever after 10 h, which persisted for 2 days. The highest recorded body temperature was 39.2°C.

**Figure 1:**
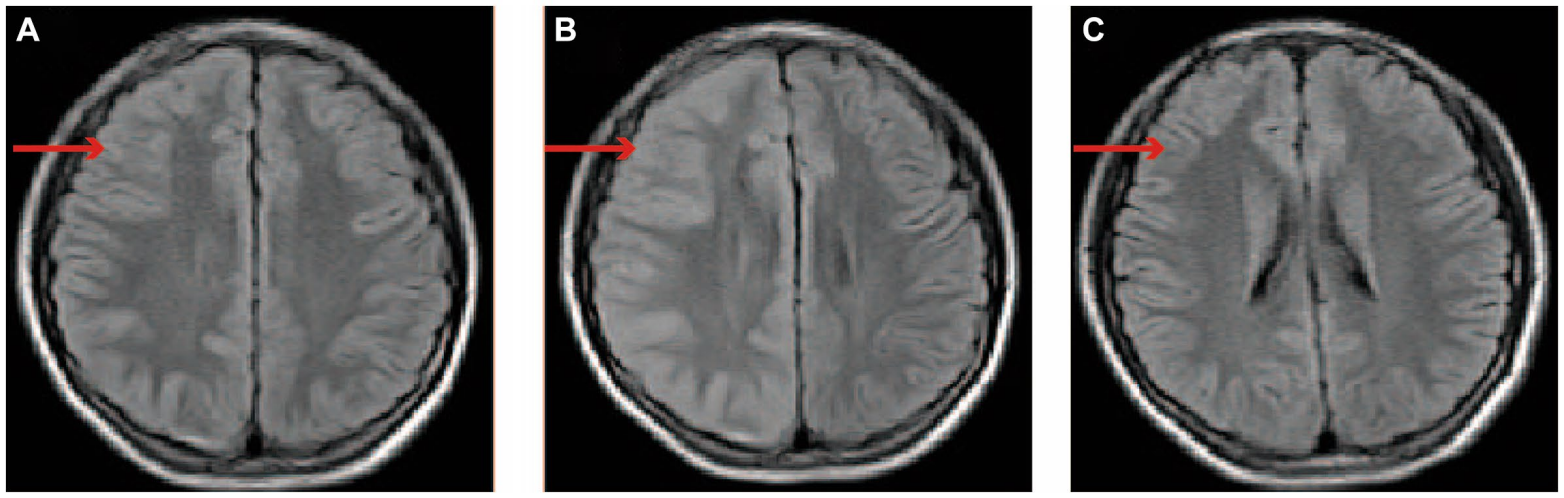
The changes of brain MRI images. **(A)** On day 1 following the onset of symptoms, the brain’s MRI displayed unremarkable findings. **(B)** On day 12 after symptom onset, the brain enhancement MRI is characterized by thickening of the cerebral cortex in the right cerebral hemisphere, the widening of the gyri, and the disappearance or shallowness of the sulci. **(C)** On day 45 after symptom onset, the MRI of the brain returned to its normal state. The red arrow indicates the right cerebral cortex. MRI: magnetic resonance imaging.

Laboratory examinations, encompassing blood routine test, C-reactive protein, and procalcitonin, exhibited no deviations. The cerebrospinal fluid (CSF) analysis and MRI of the cervical and thoracic spine (3 days after symptom onset) yielded negative findings. Computed tomography angiography (CTA) of the head and neck conducted 3 h after symptom onset, as well as brain MRI performed 22 h after symptom onset, revealed no pathological irregularities. Subsequently, an enhanced brain MRI conducted 12 days after symptom onset indicated the presence of a shallow right cerebral hemisphere groove and swelling of the gyrus. A brain MRI performed 45 days post-disease revealed normal results. EEG conducted during the boy’s hospitalization and after discharge yielded normal results. Subsequently, a genetic analysis was conducted on the male patient, revealing a heterozygous point mutation (c.1133C > T) in exon 9 of chromosome 1q23. This mutation, located at amino acid 378, results in a threonine to isoleucine (p.Thr378Ile) substitution. The imaging findings are shown in Figure 1.

Treatment and prognosis: upon admission, the boy received empirical treatment consisting of intravenous (IV) ceftriaxone, acyclovir, and intravenous gammaglobulin for acute encephalitis. He experienced a persistent headache for a duration of 14 days. By hospital day 12, the boy exhibited improved mobility, accompanied by gradual muscle strength recovery, enabling him to ambulate independently with the assistance of external objects. On the 13th day of hospitalization, he achieved autonomous ambulation without requiring external aid. Consequently, he was discharged from the hospital on the 16th day of his hospitalization. Following discharge, the boy underwent a follow-up period exceeding 3 years. During this period, he experienced multiple times headaches, the headache locations were not fixed and predominantly throbbing sensation, and persisted for a duration ranging from a few seconds to a few hours, ultimately resolving spontaneously. Additionally, the boy experienced four episodes of hemiparesis, with three instances affecting the left side and one affecting the right side, and each occurrence of hemiparesis was accompanied by concurrent headaches and fever. The hemiplegia resolved spontaneously within a short time frame of minutes to hours. However, one of the hemiplegic episodes lasted 2 days, prompting the patient’s admission to the hospital. The patient remained hospitalized for a total of 17 days, during which a brain MRI was conducted and yielded normal results. The patient gradually developed cognitive impairment and seizures, leading to a treatment regimen involving the administration of flunarizine for a period of 2 years, as well as the use of levetiracetam and Chinese traditional medicine for over 1 year.

## Discussion

ATP1A2 gene is located on chromosome 1q23 and serves as the genetic unit responsible for encoding the a2 subunit of the Na+, K + ATPase (a2NKA) (6). The a2 isoform is mainly expressed in skeletal muscle, heart, and brain, especially in astrocytes (8)_._ The missense mutation FHM2 occurs in the ATP1A2 gene, resulting in a complete or partial impairment of a2NKA function (9). This abnormality in astrocytes disrupts the clearance of extracellular K+ and glutamate, leading to a reduction in glutamate clearance and an elevation of K+ levels in the synaptic cleft. Consequently, this cascade of events triggers an augmented susceptibility to inhibitory influences throughout the cortex of the affected hemisphere, ultimately leading to cortical depolarization and the manifestation of migraine aura (10–12).

The symptoms commonly observed in individuals with FHM include reversible visual, sensory, or language disturbances, as well as varying degrees of limb hemiplegia (13). Some individuals with FHM who possess a mutation in the ATP1A2 gene have experienced severe attacks characterized by recurrent coma, fever, and/or epileptic seizures (13, 14). It is plausible to consider that the viral encephalitis and seizures diagnosed in the boy at the age of 5 may represent a manifestation of the severe acute encephalopathy associated with this disease.

FHM2 brain imaging shows biphasic cerebral blood flow changes during the prolonged aura. After approximately 18–19 h of aura symptom onset of hemiplegic migraine with prolonged aura, there might be a turning point in the transition from hypoperfusion to hyperperfusion (15). Research conducted on FHM2 mutant mice has identified heightened sensitivity of smooth muscle cells in the middle cerebral artery to changes in intracellular calcium levels, resulting in localized cerebral vasoconstriction and subsequent hypoperfusion when subjected to subthreshold stimulation. This is followed by a gradual impairment of calcium channels, opening of the blood–brain barrier, and prolonged depolarization, leading to the diffusion of water from the intracellular to the extracellular space. Following an extended depolarization period, water permeated from the cellular interior to the extracellular space, leading to a delayed occurrence of heightened perfusion (16). Brain enhancement MRI findings in patients with ATP1A2 exhibit two characteristics: (i) normal findings, primarily observed in patients with mild hemiplegic migraine (HM) (17, 18), and (ii) hypoperfusion in the initial stage of the hemisphere opposite to the hemiplegia, followed by widespread diffusion-weighted imaging hyperintense signals in the subsequent stage, often accompanied by cortical swelling in certain patients (15, 17). Regrettably, the brain CTA of the boy conducted 3 hours after the onset of symptoms yielded normal results, with no findings of hypoperfusion. A brain enhanced MRI was not conducted on the boy 22 h following the onset of symptoms, instead, solely a brain MRI was performed, which did not detect any abnormalities. Surprisingly, on the 12th day post-admission, the patient’s enhanced brain MRI revealed cortical swelling and increased cortical density, which were suggestive of hyperperfusion. In our study, it was observed that despite the patient’s recovery 2 weeks after admission, the brain MRI still exhibited a hyperperfusion image in the affected cerebral hemisphere. This phenomenon can be attributed to the persistent and long-term nature of cortical spreading depression (CSD). The significant alterations in microcirculation and metabolism induced by CSD lead to a decline in blood vessel reactivity, disruption of the neurovascular coupling effect, continuous cerebral vessel edema, and ultimately prolonged high perfusion imaging.

According to the more than three-year follow-up, the patient had several times headache and limb weakness attacks. Significantly, it has been observed that the patient experienced long periods without hemiplegia attacks from the age of 5 to 12, indicating that the duration between episodes in individuals with FHM can extend to multiple years. The occurrence of FHM episodes primarily manifests during childhood, adolescence, and early adulthood, and the presence of early severe acute encephalopathies may be an indicator of poor disease prognosis. Several neuropsychological studies have demonstrated that focal and degenerative cerebellar disorders associated with FHM can result in significant cognitive impairments (19). Furthermore, individuals with FHM2 may exhibit severe forms of intellectual disability (14, 19). Additionally, investigations have revealed that mutations in all three FHM genes have the potential to cause epilepsy, with ATP1A2 mutations being particularly prevalent (19, 20). The occurrence of recurring migraine and hemiparesis in our case during adolescence, along with the progressive emergence of epilepsy and cognitive impairments, indicates the possibility of a severe gene mutation in the boy. This case presents an opportunity to investigate the mechanisms underlying this mutated gene through animal experiments focused on FHM. Additionally, the patient requires long-term antiepileptic treatment, rehabilitation training, and ongoing follow-up.

In recent years, when considering ATP1A2 mutations, it may be necessary to consider FHM and alternating hemiplegia of childhood (AHC), as they may share the same pathological mechanisms (21–23). Diagnostic criteria for AHC include: (1) repeated episodes of hemiplegia of varying severity or duration, involving alternating sides or both sides of the body; (2) onset before 18 months of age; (3) presence of other paroxysmal clinical signs, such as dystonic posturing, choreoathetoid movements, tonic spells, nystagmus, and autonomic features; and (4) progressive cognitive and neurological decline over time (24). In our case, the child experienced predominantly left-sided hemiplegic seizures, with occasional involvement of the right side. The boy also had epilepsy and varying degrees of mental retardation in the later stages of the disease, which needed to be distinguished from ACH. In this case, the boy had a history of febrile convulsions at 6 months old, convulsions and coma at 4–5 years old, and hemiplegic migraines at 13 years old. There were no other neurological abnormalities observed in this boy for over 10 years. Additionally, only the patient’s maternal grandmother had a history of headaches, and there were no instances of hemiplegia in the family. Therefore, ATP1A2-induced hemiplegia and migraine should be carefully differentiated.

In summary, it is crucial to explore the familial history of headaches and hemiplegia in patients, even in the absence of conventional brain imaging and examination. Genetic testing is of utmost importance for individuals exhibiting signs of potential hereditary disorders.

## Data availability statement

The original contributions presented in the study are included in the article/supplementary material, further inquiries can be directed to the corresponding author.

## Ethics statement

The studies involving humans were approved by the Fifth People’s Hospital of Chengdu. The studies were conducted in accordance with the local legislation and institutional requirements. Written informed consent for participation in this study was provided by the participants’ legal guardians/next of kin. Written informed consent was obtained from the minor(s)’ legal guardian/next of kin for the publication of any potentially identifiable images or data included in this article.

## Author contributions

HZ: Writing – original draft, Writing – review & editing. LJ: Writing – review & editing, Writing – original draft. YX: Writing – review & editing. SY: Writing – review & editing, Conceptualization.
